# The Effects of Measuring Emotion: Physiological Reactions to Emotional Situations Depend on whether Someone Is Asking

**DOI:** 10.1371/journal.pone.0064959

**Published:** 2013-06-05

**Authors:** Karim S. Kassam, Wendy Berry Mendes

**Affiliations:** 1 Department of Social and Decision Sciences, Carnegie Mellon University, Pittsburgh, Pennsylvania, United States of America; 2 Department of Psychiatry, University of California San Francisco, San Francisco, California, United States of America; The University of South Wales, Australia

## Abstract

Measurement effects exist throughout the sciences–the act of measuring often changes the properties of the observed. We suggest emotion research is no exception. The awareness and conscious assessment required by self-report of emotion may significantly alter emotional processes. In this study, participants engaged in a difficult math task designed to induce anger or shame while their cardiovascular responses were measured. Half of the participants were asked to report on their emotional states and appraise their feelings throughout the experiment, whereas the other half completed a control questionnaire. Among those in the anger condition, participants assigned to report on their emotions exhibited qualitatively different physiological responses from those who did not report. For participants in the shame condition, there were no significant differences in physiology based on the self-report manipulation. The study demonstrates that the simple act of reporting on an emotional state may have a substantial impact on the body’s reaction to an emotional situation.

## Introduction

Throughout the sciences, observation is known to alter the observed. In physics, observing a particle requires another particle to interact with it, changing the properties of the particle to be observed [1]. In computer science, adding a debugger changes memory allocation, which can alter the behavior of target code. In electrical engineering, measurement of current in a circuit entails adding a voltmeter, which necessarily changes current flow. Measurement effects may also be important in emotion research–how people feel could depend on whether someone is asking. Reporting how we are feeling requires awareness and conscious assessment of our emotional states, and these processes may alter emotional experiences.

Emotions are complex processes involving multiple response channels, including physiological systems, facial and vocal expressive tendencies, and cognition [2] [3] [4]. These channels influence each other in a process that extends over time [5]: emotional events trigger sequences of neural activity [6] [7] [8], which result in changes in autonomic and neuroendocrine systems [9] [10]. Reporting how we are feeling may interrupt these processes, replacing the characteristic thoughts and appraisals of the emotion with awareness of the emotion itself. This shift of attention and change in cognition may alter other aspects of emotional response. As a result, the emotional process on which we have been asked to report could be fundamentally altered.

An assumption that emotional processes will return to normal operation following self-report is tenuous. Subjective feelings, the conscious component of emotional response, don’t arise from simple, passive perception. Awareness of our emotional states requires focal attention [11], as well as an interpretive process that tends to recruit a wide range of information [12]. Though we may have the impression that feelings simply appear in consciousness, their generation entails an active process that consumes cognitive resources [13]. Such complex cognitions are likely to interact with emotional response, which involves its own set of characteristic thoughts [14].

That conscious processing is capable of impacting emotion is well documented. Detailed, causal analyses of past emotional experiences can have a significant impact on current emotional states. Writing about negative emotional experiences for a few minutes every day can lead to significant physical and mental health improvements [15] [16]. Talking about past emotional experiences can also affect present physiology, with emotional disclosure being associated with changes in skin conductance levels and cardiovascular physiology [17] [18]. Attempting to understand the causes of negative emotion thus serves to ameliorate their negative effects. Reflective reasoning can also mitigate the negative effects of ongoing emotional experiences. Reappraising an emotional situation to be less impactful (e.g. consciously adopting the mindset of a medical student when watching a disgusting video) decreases self-reported emotional intensity, reduces emotion-related expressive behavior, and moderates a number of physiological variables [19] [20].

Though diary writing and reappraisal require substantial cognitive processing, more subtle manipulations of awareness may also impact emotional processing. Scheier and Carver argue that awareness can enhance the saliency of emotion and result in a change in the relationship between emotion and behavior [21]. They used individual differences to measure awareness and strategically placed mirrors to manipulate awareness, and found awareness of anger led to more aggressive behavior [22], awareness of fear led to reluctance in approaching a snake and in receiving an electric shock [23] [24], and awareness of positive and negative emotions lead to changes in ratings of emotional images [25]. More recently, simply labeling the emotional content of an image has been found to affect neural activity. Participants who label emotions in faces shown to them exhibit reduced amygdala activity relative to those in control conditions [26] [27], suggesting a reduction in the emotional impact of those stimuli. Given the variety of awareness prompts that have been found to affect different aspects of emotional processing, we hypothesized that simply asking participants how they are feeling on various dimensions might affect their emotional reactions.

We measured participants’ cardiovascular responses during a difficult mental arithmetic task designed to elicit anger (a non self-conscious emotion), shame (a self-conscious emotion) or no emotion (i.e. a control condition; [28] [29] [30]. We provide a discussion as to the identifiability and discriminability of the emotion inductions in the general discussion below). Previous research has shown that such protocols result in distinct cardiovascular profiles; relative to shame, anger is associated with larger increases in cardiac output (a volume based measure of oxygenated blood pumped by the heart) and lower vascular resistance [31] [32] [30]. These patterns of cardiovascular reactivity allow for a putative distinction between emotional experiences without the need to ask participants for a subjective report. Self-report of emotion instead served as a second independent variable. We manipulated participants’ thoughts and feelings by having them rate their emotional state along several dimensions, as well as appraise their situations. Half of the participants were asked to report on their emotional states and appraisals at various times throughout the experiment, while the other half were asked control questions that did not require conscious assessment of their emotional state. We hypothesized that asking participants to report their emotions could change their physiological responses.

## Methods

One hundred and twelve residents of the Cambridge, MA area (67 females, *M*_age_ = 23.4, *SD* = 4.0) participated in exchange for $15. Participants were recruited through online classifieds and were prescreened prior to scheduling their lab visit. We excluded participants if they had a history of diagnosed depression or anxiety, had a doctor-diagnosed heart murmur/arrhythmia, or were pregnant. Participants were randomly assigned to one of 6 conditions in a 2×3 design including 2 levels of the report factor (*report* vs. *no-report*), and 3 levels of the emotion factor (*anger* vs. *shame* vs. *control*).

### Procedure

After application of physiological sensors, participants sat quietly for a 5-minute baseline period. Then, those in the *report* condition were asked to rate how *proud, annoyed, happy, self-conscious, frustrated, sad, enthusiastic, calm, embarrassed, excited, angry, alert, ashamed, and satisfied* they felt, each on an analog scale anchored with endpoints “Not at all” and “A great deal.” They also rated the degree to which they disagreed or agreed to ten statements: *I feel confident about my abilities, I am having trouble understanding the instructions given to me, I feel impatient with others, I feel that others respect and admire me, I want to argue with others, I am easily distracted by others, I feel uncomfortable when no one is speaking to me, I feel small, I can calm down easily,* and *I am losing my temper*. For the *no report* condition we created a questionnaire that was neither emotional nor self-reflective in nature. Instead, in the *no report* condition participants answered the same number of questions about their technology use (e.g., “How often do you use a webcam?”) using the same response scales.

Following the self-report, participants completed a difficult arithmetic task either through prompts from a computer while alone in a room (in the control condition) or in the presence of an evaluator (in the anger and shame conditions). All participants were asked to count backwards in steps of seven or thirteen from five-digit numbers. (In order to maintain a steady level of difficulty for all participants, those who were able to countdown by more than 119 (i.e. 17 steps) in the first minute were asked to count in steps of 13 for subsequent iterations). In both emotion conditions (anger and shame), evaluators gave negative feedback after each performance block, implying that the participant was performing poorly on the counting task and that excessive movement was interfering with physiological signals. We manipulated anger and shame by changing the tone in which the experimenter delivered the feedback. In the anger condition, evaluators were trained to deliver the negative feedback in a way that made them sound annoyed and incompetent (using the technique described by [29]). In the shame condition, the evaluators gave the same negative feedback, but in a tone that was warm and matter-of-fact. The warmth of the experimenter in the shame condition was intended to cause participants to like the experimenter, making it more difficult to discount the negative performance feedback. The emotion conditions were designed so that participants would attribute their poor performance in the task either externally to an unreasonable experimenter (anger condition), or internally, to themselves (shame condition), thus activating the core relational themes of the two emotions [33] [35] [36]. Feedback was given over an intercom in order to minimize differences between the two conditions. In the control condition, participants completed the task through prompts from a computer program and did not receive negative feedback.

After this manipulation, participants in the *report* condition completed a second emotion questionnaire identical to the one they had completed previously, and those in the *no report* condition completed another control questionnaire. We then introduced a second task–a digit span task [34]. In each of 14 trials, the experimenter read a series of digits and asked participants to repeat those digits in reverse order. The number of digits started at two for the first trial and increased by one digit every other trial. During this time, the experimenter spoke in a neutral tone and did not provide any feedback. This period thus provided a window in which to analyze critical physiological measures, immediately following both manipulations, and during which emotion was not concurrently being manipulated [35] [17].

To conclude the experiment, participants in the emotion conditions also rated the degree to which they agreed or disagreed with the statements “The experimenter did his/her job professionally’, ‘I enjoyed my interaction with that experimenter’ and ‘I think the experimenter is a likable person’ each on continuous analog scales coded on a range from −100 to 100 and anchored with the terms ‘Strongly Disagree’ and ‘Strongly Agree.’

### Physiological responses

Our primary physiological variables of interest were heart rate (HR), pre-ejection period (PEP), cardiac output (CO, the total volume of blood pumped by the heart per minute), and total peripheral resistance (TPR, an overall measure of vasodilation versus vasoconstriction in the arterioles). The first two measures provide indications of sympathetic nervous system activation (especially PEP), and the latter two provide distinctions between approach (i.e., challenge) and withdrawal (i.e., threat) motivational states and have been linked to anger and shame [23] [28] [30].

These measures were collected or derived from electrocardiography (Biopac ECG module, Goleta, CA), impedance cardiography (HIC-2000, Bio-Impedance Technology Inc., Chapel Hill, NC), and blood pressure measurement (Colin Prodigy, Colin Medical Instruments, San Antonio, TX), and integrated using the Biopac MP 150. Heat rate was obtained using two spot sensors in a lead II configuration (right arm, left leg). Impedance cardiography utilized four adhesive mylar bands that completely encircled the participant’s neck and torso. ECG and ICG signals were sampled at 1000 Hz. All data were visually inspected, artifacts were edited and then responses averaged in one-minute bins using Mindware software (Lafeyette, OH). All measures were continuous with the exception of blood pressure (BP), which was taken intermittently at pre-defined intervals, as frequent BP measurement might artificially elevate BP levels. Intermittent measurement of BP resulted in some data loss.

### Behavioral coding

Because participant self-reports were used as one of the independent variables, we include observations of participants’ behavior as one of the emotion manipulation checks. Two coders, naïve to experimental condition, watched videos of each participant during both the mental arithmetic and digit span tasks and rated how *Annoyed, Frustrated, Angry, Hostile, Impatient, Agitated, Dominant, Self-conscious, Embarrassed, Ashamed, Sad, Submissive,* and *Disengaged* the participant appeared, each on five point scales. These videos did not include audio.

### Analysis strategy

We first tested for baseline differences of physiological responses prior to random assignment to condition. We then calculated reactivity scores (subtracting the last minute of the baseline period from the average of the two task period minutes). Next, all dependent measures were subjected to a 2×3 (or 2×2, when appropriate) between subjects ANOVA. When significant effects were observed, uncorrected (Fischer’s LSD) comparisons between conditions were examined.

## Results

### Data Loss

Four participants were excluded because they withdrew and/or failed to carry out instructions, and data from six participants could not be analyzed due to equipment/sensor failure or electrical artifacts. This left a total of 102 participants distributed across conditions as follows: 15 in *no-report*/*control*, 18 in *no-report/anger*, 19 in *no-report/shame*, 18 in *report/control*, 17 in *report/anger*, and 15 in *report/shame*.

### Manipulation Checks

We first examined the effectiveness of our anger and shame manipulations using evaluations of the experimenter’s behavior obtained at the conclusion of the experiment. We found main effects of *emotion condition* on all three questions. Relative to those in the *shame* condition, participants in the *anger* condition saw the experimenter as less professional (*M*_Anger_ = −10.5, *SE* = 10.9, *M*_Shame = _47.5, *SE* = 11.3, *F*(1, 65) = 13.68, p<.001), less likable (*M*_Anger_ = −9.5, *SE* = 9.6, *M*_Shame = _47.1, *SE* = 9.8, *F*(1, 65) = 17.02, p<.001), and enjoyed their interaction with her less (*M*_Anger_ = −16.8 *SE* = 10.5, *M*_Shame = _29.8, *SE* = 10.9, *F*(1, 65) = 9.45, p = .003). As intended, our anger manipulation produced more negative reactions to the experimenter than the shame manipulation. There were no effects of the other manipulation, self-report, nor any interaction effects on any of these measures (all *p*’s>.3).

### Behavioral Coding

Next we analyzed the results of behavioral coding. We averaged coder’s ratings and combined the *Annoyed, Frustrated, Angry, Hostile, Impatient, Agitated,* and *Dominant* ratings into a single *Anger* measure (Cronbach’s α = 0.79), and the *Self-Conscious*, *Embarrassed, Ashamed, Sad,* and *Submissive* ratings into a single *Shame* measure (α = 0.87). The *Disengaged* ratings did not correlate well with either measure. We present the average scores here for simplicity. Factor analysis yields a similar dichotomy, with the exception that *Frustration* loaded on both anger and shame factors (details of the factor analysis results are available upon request).

During the digit span task, those in the anger condition appeared significantly more angry (*M* = 1.59, *SE* = .09) than those in the neutral condition (*M* = 1.19, *SE = *.09; *t*(96) = 3.28, p = .002); the difference in anger behavior between anger and shame conditions (*M* = 1.46, *SE* = .09) failed to reach statistical significance (*t*(96) = 1.11, p = .27). Participants in the shame condition appeared significantly more ashamed (*M* = 2.08, *SE* = .16) than those in the neutral condition (*M* = 1.28, *SE* = .17; *t*(96) = 3.47, p = .001); the difference in shame behavior between shame and anger conditions (*M* = 2.11, *SE* = .15) failed to reach statistical significance (*t*(96)<1). There were no significant effects of report, and no interactions.

### Baseline

We first analyzed physiological responses in the fifth (and final) minute of the baseline period (i.e., when participants had been resting for the maximum period of time), prior to any experimental manipulation. No significant differences were observed in any of our physiological measures of interest at baseline: HR (emotion main effect: *F*(2, 96) = 2.00, *p* = .14; report main effect: *F*(2, 96)<1; interaction: *F*(2, 96)<1), PEP (emotion: *F*(2, 96)<1, report: *F*(1, 96) = 2.34, *p* = .13, interaction: *F*(2, 96)<1), CO (all *F*’s<1), TPR (all *F*’s<1), systolic blood pressure (emotion: *F*(2, 92) = 1.28, *p* = .28, report: *F*(1, 92)<1, interaction: *F*(2, 92) = 1.78, *p = *.17), and diastolic blood pressure (emotion: *F*(2, 92) = 1.87, *p* = .16, report: *F*(1, 92)<1, interaction: *F*(2, 92)<1).

### Cardiovascular Reactivity

To examine the question of whether the emotion induction, the report manipulation, or their interaction influenced physiological responses, we examined changes in cardiovascular reactivity during the digit span task. We created reactivity scores by subtracting baseline values from values obtained from the digit span period.

As in previous research [9] [37] [30] heart rate showed a significant main effect of emotion, *F*(2, 96) = 20.61, *p*<.001, such that those in the anger condition (*M* = 15.51, *SE* = 1.34) had a greater increase in HR than those in the shame condition (*M* = 10.13, *SE* = 1.41; *t*(96) = 2.75, *p* = .007); who in turn had a greater increase in HR than those in the control condition (*M* = 3.05, *SE* = 1.41; *t*(96) = 3.55, *p = *.001). HR also showed a significant main effect of report, *F*(1,96) = 4.21, *p* = .043), such that those in the emotion-report condition (*M* = 7.92, *SE* = 1.16) had a smaller increase in HR than those in the no-report condition (*M* = 11.21, *SE* = 1.11). There was no significant emotion by report interaction, *F* (2, 96) = 1.53, *p* = .221.

Pre-ejection period, a measure of sympathetic activation [38], also showed a significant main effect of emotion, F(2, 96) = 12.76, p<.001. Those in the anger condition (*M* = −11.29, *SE* = 1.20) showed a greater decrease in PEP from baseline than did those in the shame condition (*M* = −5.51, *SE* = 1.26; *t*(96) = 3.31, *p* = .001), who in turn showed a marginally greater decrease than those in the control condition (*M* = −2.42, *SE* = 1.26; *t*(96) = 1.74, *p* = .086). There was no significant main effect of report, F(1, 96) = 1.50, p = .223; and no interaction, F(2, 96)<1.

**Figure 1:**
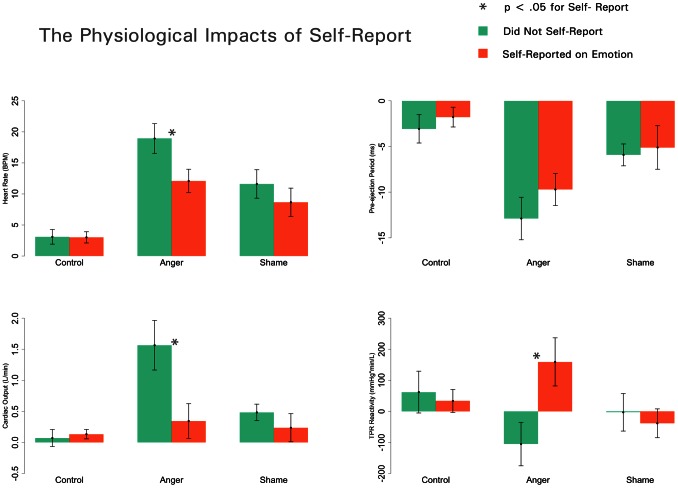
Cardiac output (CO) and total peripheral resistance (TPR), heart rate (HR), and pre-ejection period (PEP) reactivity vary by emotion induction and self-report. Values represent changes from baseline; error bars indicate standard error.

We then turned to measures of cardiovascular reactivity that have differentiated shame and anger in past research [28] [23] [30]. Cardiac output, a measure of cardiac efficiency, showed a significant main effect of emotion, *F*(2, 96) = 6.59, *p* = .002, such that those in the anger condition (*M = *.96, *SE* = .17) had greater CO increase than those in the shame (*M* = .36, *SE* = .18; *t*(96) = 2.45, *p* = .016). The control condition (*M* = .10, *SE* = .18) differed significantly from the anger condition (*t*(96) = 3.53, *p* = .001), but not the shame condition (*t*(96) = 1.04, *p* = .302). CO also showed a significant main effect of report, *F*(1, 96) = 4.73, p = .032, such that those in the *no report* condition (*M* = .71, *SE = *.14) showed greater CO than those in the *report* condition (*M* = .24, *SE* = .14). However, these effects were qualified by a significant interaction, *F*(2, 96) = 4.36, *p* = .015. Simple effects tests confirmed that among participants in the anger induction, reporting on one’s emotional state was associated with lower CO (*M* = .35, *SE = *.32) than not reporting (*M* = 1.74, *SE* = .44; *t*(96) = 3.64, *p*<.001). In contrast, the emotion-report manipulation had no influence on CO responses among participants in the shame or control conditions, (*t*’s<1; see Figure 1).

TPR did not exhibit main effects for emotion, *F*(2, 65)<1; or report, *F*(1, 65) = 1.762, *p* = .19, but did yield a significant interaction, *F*(2, 65) = 3.86, *p* = .026. In line with the CO data, participants in the anger induction who reported on their emotional states showed higher TPR, (*M* = 159.3, *SE* = 63.2) than participants who did not report (*M* = −105.4, *SE* = 60.54; *t*(65) = 3.02, *p = *.004). TPR was not affected by the emotion-report manipulation for those assigned to the shame or control conditions (*t*’s<1; Figure 1). In sum, self-reports of emotional states resulted in qualitatively different patterns of cardiovascular responses during anger but not shame induction.

TPR is a precursor to hemodynamic changes, and thus represents a more proximal measure of emotion-related physiological changes than blood pressure. However, because a considerable body of research on the physiological concomitants of anger focuses on blood pressure [39] [40] [41], we also examined individual blood pressure parameters. Systolic blood pressure showed a significant main effect of emotion *F*(2, 65) = 8.13, *p* = .001, such that those in the anger condition showed greater increases from baseline (*M* = 13.23, *SE* = 1.87) than those in the shame (*M* = 10.06, *SE* = 1.82, *t*(65) = 2.82, *p* = .006) and control (*M* = 2.46, *SE* = 1.99, *t*(65) = 3.95, *p*<.001) conditions. The *shame* and *control* conditions did not differ significantly (*t*(65) = 1.21, *p* = .23). Neither report (*F*(2,65)<1), nor the report by emotion interaction (*F*(2,65)<1) had a significant effect. Diastolic blood pressure showed a marginal main effect of emotion *F*(2, 65) = 2.53, *p* = .087. Those in the anger condition showed significantly greater increases from baseline (*M* = 7.94, *SE* = 1.57) than those in the shame condition (*M* = 3.24, *SE* = 1.53, *t*(65) = 2.14, *p* = .036) and marginally greater increases than those in the control condition (*M* = 4.15, *SE* = 1.67, *t*(65) = 1.66, *p* = .10). The shame and control conditions did not differ significantly (*t*(65)<1). Neither report (*F*(2,65)<1), nor the report by emotion interaction (*F*(2,65) = 1.24, *p* = .30) had a significant effect.

## Discussion

Instruments of observation frequently alter the observed, and self-reports of emotional experience are no exception. We induced emotion in participants using an evaluative math task, and found that the physiological responses of participants in the anger condition–but not the shame condition–were qualitatively different when they were asked to report on their emotions. Participants in the anger condition showed larger increases in CO when they were not reporting their emotions than when they were reporting. In contrast, for those in the shame and control conditions, reporting did not result in significant differences in CO. Participants in the anger condition also showed a decrease in TPR (i.e. vasodilation) when they were not reporting on their emotions, and an increase in TPR (i.e. vasoconstriction) when they were reporting. Self-reports of emotion did not lead to significant differences in TPR for participants in the shame or control conditions. Overall, our results show that asking people about their emotions can have a significant impact on their physiological responses, and that the impact may depend on the type of emotion experienced.

### Rumination as Possible Mechanism

Self-reports of emotional experience require introspection and entail an awareness that may change emotional processes. Awareness also opens the door to maladaptive conscious intervention, i.e. rumination, which could likewise lead to changes in physiological response. Self-focused attention goes hand in hand with rumination [42], and when participants adopt a first-person perspective to analyze their feelings, as is frequently encouraged by self-report measures (e.g. “How do you feel right now?”), negative consequences may follow [43] [44]. A wealth of literature on rumination demonstrates that consciously and repeatedly thinking about negative events can facilitate anti-social behavior [45], hamper problem solving [42], and lead to cardiovascular disease and psychopathology [46] [47] [48] [49]. In terms of physiological responses, rumination has been found to increase blood pressure [50], and prolong and amplify cortisol responses [51]. These changes are typical of physiological threat reactivity [52], a stress response characterized in part by decreases in cardiac output (CO) and increases in total peripheral resistance (TPR). In our experiment, participants in the anger condition who were asked to report on their emotions showed precisely these effects, suggesting that rumination may have played a role.

### Differences between Emotion Manipulations

Rumination may also provide an explanation for why the self-report manipulation affected participants in the anger and not the shame condition. Provoked individuals are especially prone to rumination [53]. Moreover, anger inductions are typically associated with increases in heart rate and CO and decreases in TPR [54] [37] [29] [30] effects that rumination can counteract or reverse. Shame, on the other hand, is a self-conscious emotion, by definition requiring awareness as well as elaborate cognitive processes and a self-evaluative capacity [55] [56] [57]. Shame naturally evokes rumination [58] [59], reducing the potential impact of emotional awareness. Self-report may have had little effect on shame because shame naturally involves the mechanisms by which manipulations of awareness operate.

### What about Other Emotions?

More generally, the presence and quality of measurement effects appear to vary across emotions. In our study, report and appraisal resulted in a qualitative change in physiological response in the anger condition but not in the shame condition. Existing literature suggests that other negative emotions might be significantly affected by measurement through self-report, and that those changes may be beneficial. Research by Lieberman and colleagues [26] [60], shows that bringing awareness to negative states, whether by labeling the emotion or describing the emotional stimuli, significantly reduces neural activation related to and subsequent self-reports of distress and fear as well as anger.

Measurement effects may also affect positive emotions. Labeling the content of positive pictures has resulted in reduced self-reports of pleasure [60], and the frequency of happiness reports has been found to reduce subsequent reported happiness among participants high in neuroticism or depression [61]. Similarly, subtle interventions that highlight why positive events occur serve to reduce happiness resulting from those positive events [62]. Awareness manipulations can sometimes have beneficial effects when it comes to positive emotions, however. Over longer time periods, expressions of gratitude have been found to increase subjective well-being [63]. How measurement effects change emotional experience, as well as which emotions are impacted, remain important questions for future research.

### Physiology of Anger

Given the qualitative differences observed across the report and no-report conditions of the anger manipulation, one natural question that follows is which physiological response is most representative of anger?

The question assumes at least some emotion specificity of physiological responses, an assumption that has received modest support [64] [54] [65] [37] [9]. Anger inductions have typically been associated with increases in heart rate and CO [54] [37] [29] [30]. These results would suggest that the physiological responses exhibited by those in the no-report condition were more representative of anger. However, few researchers have collected CO and TPR data in the context of an anger manipulation and many have had participants report on their emotional states, making a definitive conclusion difficult to draw.

Rather than pointing to a single, invariant anger physiology, the results may be taken to indicate multiple physiological profiles of anger. Research suggests that *anger* and *anger with rumination* have distinct physiological responses. In one experiment, participants asked to recall an unresolved anger experience and ruminate over it showed increased peripheral vasoconstriction relative to those in a recall and reappraise condition [66]. In another experiment, participants angered by a difficult task and unreasonable experimenter showed higher systolic blood pressure when they were asked to write about the provocation [67].

A similar distinction between *internalized* and *externalized* anger has frequently been made by clinical and health psychologists [68]. Each construct is capable of making unique predictions: internalized anger independently predicts depressive symptoms, and externalized anger uniquely predicts hostility [69] [70]. Recent theory regarding the health consequences of stress [46] likewise supports this distinction, suggesting that rumination is a key factor that allows stress to exude deleterious effects on the cardiovascular system. Our results provide evidence of a mechanism for this pathway. When angry participants were asked to report on their emotional state, they showed increases in TPR, which can limit oxygenated blood flow to the periphery. Participants made aware of their emotional state thus showed a stress response consistent with *threat* [35], which has previously been implicated as a precursor for cardiovascular disease [71]. Differences in CO across the conditions may likewise hold important health implications. Recent research has shown that higher levels of CO in older age are associated with decelerated brain aging and lower risk of Alzheimer’s disease [72]. Importantly, we are not implying that a cardiac response is inherently healthy or that a vascular response is inherently unhealthy. Indeed, a cardiac response that lingers without recovering can be detrimental [71]. What our data do suggest is that the physiological differences observed in the anger conditions are of practical as well as theoretical import.

### Limitations and Alternative Explanations

One limitation of the present study is that not all manipulation checks were able to differentiate the anger and shame conditions. Evaluations of the experimenter differed between the two emotion conditions, as did physiological response, and both are consistent with an anger and shame interpretation. Behavioral coding suggested that anger and shame differed from the control condition in consistent ways, but behavioral coding did not differentiate between anger and shame conditions. We chose to examine anger and shame because we could induce the pair with only a subtle change in manipulation, reducing the potential confound created by vastly different emotion manipulations. In doing so, we necessarily diminished the difference between the two manipulations. As a result, it is likely that some participants in our anger condition felt some shame, and that some participants in our shame condition felt some anger. The behavioral coding results may also reflect the fact that anger and shame are closely related emotions [73]. Not only can the experience of shame lead one to anger [74], but also the experience of anger can lead one to shame [75].

The present research represents one of the first attempts to examine the effects of subjective reports on physiological reactivity stemming from an emotional experience, and many questions remain. Our approach, which investigated two emotions each with a single manipulation, cannot fully determine when emotions will be affected by report manipulations, and when they will not. Anger and shame differ in that anger is not a self-conscious emotion whereas shame is, but there are other differences between the emotions that might account for the differences observed. For example, reporting one’s anger may comprise a greater violation of social desirability, resulting in an increase in threat stress response.

Another possibility is that shame did not show an effect of report because the shame manipulation was relatively weak. Identifying emotion strength with change in a single physiological measure – for example, heart rate – would imply that our manipulation of shame was weaker than our manipulation of anger. Such an inference, however, is fraught with theoretical issues [9] [64]. In contrast to the physiological indicators, both self-reports and behavioral coding suggest that participants in the shame condition felt as much if not more emotion than those in the anger condition. These variables, however, require comparison on different response scales. In the end, as with any comparison of different emotions, it is difficult to conclude that either anger or shame manipulation was more effective.

Nor is it possible to conclusively identify rumination as the key mechanism whereby these effects occur. We favor rumination as a likely mechanism because it is consistent with both the differences in the effect of the self-report manipulation observed between anger and shame conditions, and with the specific physiological changes observed in anger. But we did not test for rumination directly. Another possible mechanism is that self-reports and appraisals serve to contextualize the emotion and thereby change the experience. That is, theories suggest that contextualization is a key component of emotional processes [76] [77]. In making participants aware of their thoughts and feelings, self-reports may have served to change this aspect of emotional response. Though contextualization does not provide a clear prediction for differences between emotions, it nevertheless provides another avenue whereby self-reports may alter physiology.

Finally, our preliminary investigation leaves open the question of whether self-reports of emotion, self-reports of our additional statements, or both are necessary to shift physiological response in anger. We included both emotion reports and appraisals in our self-report questionnaire in order to force participants to introspect on their feelings. As a result, the present data do not allow us to tease their effects apart.

### Conclusion

What we can conclude is that the act of reporting on emotional states can alter emotional response. Even the simplest verbal measures invoke self-awareness of psychological processes [60], causing changes in the neural and physiological concomitants of emotion. When emotion manipulations are interrupted by self-report measures, thoughts that are characteristic of emotional response are likely to be replaced by awareness and introspection. As these thoughts change, so too does physiology, fundamentally altering the emotional response.

## References

[1] Feynman RP, Leighton RB, Sands N (1971) The Feynman lectures on physics (Vol 3).

[2] Barrett LF, Bliss-Moreau E (2009) Affect as a psychological primitive. Adv Exp Soc Psychol 41: 167–218. 20552040 10.1016/S0065-2601(08)00404-8 PMC2884406

[3] Keltner D, Lerner JS (2010) Emotion. In: Gilbert DT, Fiske ST, Lindsay G, editors. The handbook of social psychology. New York: McGraw Hill. 312–347.

[4] Larsen RJ, Fredrickson BL (1999) Measurement issues in emotion research. In: Kahneman D, Diener E, Schwartz N, editors. Well-being: The foundations of hedonic psychology. New York: Gilford. 40–60.

[5] Scherer K (2005) What are emotions? And how can they be measured? Semiotica 44: 695–729.

[6] Northoff G, Richter A, Gessner M, Schlagenhauf F, Fell J, et al (2000) Functional dissociation between medial and lateral prefrontal cortical spatiotemporal activation in negative and positive emotions: A combined fMRI/MEG study. Cereb Cortex 10: 93–107. 10639399 10.1093/cercor/10.1.93

[7] Carreti L, Martin-Loeches M, Hinojosa JA, Mercado F (2001) Emotion and attention interaction studied through event-related potentials. J Cogn Neurosci 13: 1109–1128. 11784449 10.1162/089892901753294400

[8] Yamasaki H, LaBar KS, McCarthy G (2002) Dissociable prefrontal brain systems for attention and emotion. Proc Nat Acad Sci, USA 99: 11447–51. 12177452 10.1073/pnas.182176499 PMC123276

[9] Larsen JT, Berntson GG, Poehlmann KM, Ito TA, Cacioppo JT (2008) The psychophysiology of emotion. In: Lewis M, Haviland-Jones JM, Barrett LF, editors. The Handbook of Emotion. New York: Russell Sage Foundation. 180–195.

[10] Lovallo WR, Thomas TL (2000) Stress hormones in psychophysiological research: Emotional, behavioral, and cognitive implications. In: Cacioppo JT, Tassinary LG, Berntson G, editors. Handbook of psychophysiology. New York: Cambridge University Press. 342–367.

[11] Lambie JA, Marcel AJ (2002) Consciousness and the varieties of emotion experience: A theoretical framework. Psychol Rev 109: 219–259. 11990318 10.1037/0033-295x.109.2.219

[12] Robinson MD, Clore GL (2002) Belief and feeling: Evidence for an accessibility model of emotional self-report. Psychol Bull 128: 934–960. 12405138 10.1037/0033-2909.128.6.934

[13] Kron A, Schul Y, Cohen A, Hassin R (2010) Feelings don’t come easy: Studies on the effortful nature of feelings. J Exp Psychol Gen 139: 520–534. 20677897 10.1037/a0020008

[14] Leventhal H, Scherer K (1987) The relationship of emotion to cognition: A functional approach to a semantic controversy. Cogn Emot 1: 3–28.

[15] Pennebaker JW (1997) Writing about emotional experiences as a therapeutic process. Psychol Sci 8: 162–166.

[16] Frattaroli J (2006) Experimental disclosure and its moderators: A meta-analysis. Psychol Bull 132: 823–865. 17073523 10.1037/0033-2909.132.6.823

[17] Mendes WB, Reis H, Seery M, Blascovich J (2003) Cardiovascular correlates of emotional expression and suppression: Do content and gender context matter? J Pers Soc Psychol 84: 771–792. 12703648 10.1037/0022-3514.84.4.771

[18] Hughes CF, Uhlmann C, Pennebaker JW (1994) The body's response to processing emotional trauma: Linking verbal text with autonomic activity. J Pers 62: 565–585. 7861305 10.1111/j.1467-6494.1994.tb00309.x

[19] Gross JJ, Levenson RW (1995) Emotion elicitation using films. Cogn Emot 9: 87–108.

[20] Gross JJ (2002) Emotion regulation: Affective, cognitive, and social consequences. Psychophysiology 39: 281–291. 12212647 10.1017/s0048577201393198

[21] Carver CS, Scheier MF (1982) Control theory: A useful conceptual framework for personality–social, clinical, and health psychology. Psychol Bull 92: 111. 7134324

[22] Scheier MF (1976) Self-awareness, self-consciousness, and angry aggression1. J Pers 44: 627–644. 1011070 10.1111/j.1467-6494.1976.tb00142.x

[23] Carver CS, Blaney PH, Scheier MF (1979) Focus of attention, chronic expectancy, and responses to a feared stimulus. J Pers Soc Psychol 37: 1186. 490310 10.1037//0022-3514.37.7.1186

[24] Scheier MF, Carver CS (1977) Self-focused attention and the experience of emotion: Attraction, repulsion, elation, and depression. J Pers Soc Psychol 35: 625. 909041 10.1037//0022-3514.35.9.625

[25] Scheier MF, Carver CS, Gibbons FX (1981) Self-focused attention and reactions to fear. J Res Pers 15: 1–15.

[26] Lieberman MD, Eisenberger NI, Crockett MJ, Tom SM, Pfeifer JH, et al (2007) Putting feelings into words: Affect labeling disrupts amygdala activity to affective stimuli. Psychol Sci 18: 421–428. 17576282 10.1111/j.1467-9280.2007.01916.x

[27] Creswell JD, Way BM, Eisenberger NI, Lieberman MD (2007) Neural correlates of dispositional mindfulness during affect labeling. Psychosom Med 69: 560–565. 17634566 10.1097/PSY.0b013e3180f6171f

[28] Jamieson JP, Koslov K, Nock MK, Mendes WB (2013) Experiencing discrimination increases risk-taking. Psychol Sci 24: 131–139. 23257767 10.1177/0956797612448194

[29] Mauss IB, Cook CL, Cheng JYJ, Gross JJ (2007) Individual differences in cognitive reappraisal: Experiential and physiological responses to an anger provocation. Int J Psychophysiol 66: 116–124. 17543404 10.1016/j.ijpsycho.2007.03.017

[30] Mendes WB, Major B, McCoy S, Blascovich J (2008) How attributional ambiguity shapes physiological and emotional responses to social rejection and acceptance. J Pers Soc Psychol 94: 278–291. 18211177 10.1037/0022-3514.94.2.278 PMC2535927

[31] Herrald MM, Tomaka J (2002) Patterns of emotion-specific appraisal, coping, and cardiovascular reactivity during an ongoing emotional episode. J Pers Soc Psychol 83: 434–450. 12150239

[32] Jamieson JP, Nock MK, Mendes WB (2012) Mind over matter: Reappraising arousal improves cardiovascular and cognitive response to stress. J Exp Psychol Gen 141: 417–422. 21942377 10.1037/a0025719 PMC3410434

[33] Tracy JL, Robins RW (2006) Appraisal antecedents of shame and guilt: Support for a theoretical model. Pers Soc Psychol Rev 32: 1339–1351. 10.1177/0146167206290212 16963605

[34] Wechsler D (2003) Wechsler Intelligence Scales for Children Fourth Edition (WISC-IV). San Antonio, TX: Psychological Corporation.

[35] Blascovich J, Mendes WB (2010) Social psychophysiology and embodiment. In: Fiske ST, Gilbert DT, editors. The Handbook of Social Psychology. New York: McGraw Hill. 194–227.

[36] Weiner B (1980) The role of affect in rational (attributional) approaches to human motivation. Educ Res 9: 4–11.

[37] Stemmler G (1989) The autonomic differentiation of emotions revisited: Convergent and discriminant validation. Psychophysiology 26: 617–632. 2629011 10.1111/j.1469-8986.1989.tb03163.x

[38] Brownley KA, Hurwitz BE, Schneiderman N (2000) Cardiovascular psychophysiology. In: Cacioppo JT, Tassinary LG, Berntson GG, editors. Handbook of psychophysiology. Cambridge: Cambridge University Press. 224–264.

[39] Chesney MA, Rosenman RH (1985) Anger and hostility in cardiovascular and behavioral disorders; Anger and hostility in cardiovascular and behavioral disorders. Washington: Hemisphere Publishing Corporation.

[40] Spielberger CD, Johnson EH, Russell SF, Crane R, Jacobs GA, et al (1985) The experience and expression of anger: Construction and validation of an anger expression scale. Aggr Behav 11: 5–30.

[41] Suls J, Wan CK, Costa PT (1995) Relationship of trait anger to resting blood pressure: A meta-analysis. Health psychol 14: 444. 7498116 10.1037//0278-6133.14.5.444

[42] Lyubomirsky S, Nolen-Hoeksema S (1995) Effects of self-focused rumination on negative thinking and interpersonal problem-solving. J Pers Soc Psychol 69: 176–190. 7643299 10.1037//0022-3514.69.1.176

[43] Ayduk O, Kross E (2008) Enhancing the pace of recovery: Differential effects of analyzing negative experiences from a self-distanced vs. self-immersed perspective on blood pressure reactivity. Psychol Sci 19: 229–231. 18315794 10.1111/j.1467-9280.2008.02073.x

[44] Kross E, Ayduk O (2011) Making meaning out of negative experiences by self-distancing. Curr Dir Psychol Sci 20: 187–191.

[45] Bushman BJ, Baumeister RF, Phillips CM (2001) Do people aggress to improve their mood? Catharsis beliefs, affect regulation opportunity, and aggressive responding. J Pers Soc Psychol 81: 17–32. 11474722

[46] Gerin W, Zawadzki MJ, Brosschot JF, Thayer JF, Christenfeld N, et al.. (2012) Rumination as a mediator of chronic stress effects on hypertension: A causal model. Int J Hypertens 2012. 10.1155/2012/453465 PMC3296188 22518285

[47] Brosschot JF, Gerin W, Thayer JF (2006) The perseverative cognition hypothesis: A review of worry, prolonged stress-related physiological activation, and health. J Psychosom Res 60: 113–124. 16439263 10.1016/j.jpsychores.2005.06.074

[48] Nolen-Hoeksema S (2000) The role of rumination in depressive disorders and mixed anxiety/depressive symptoms. J Abnorm Psychol 109: 504–511. 11016119

[49] Treynor W, Gonzalez R, Nolen-Hoeksema S (2003) Rumination reconsidered: A psychometric analysis. Cognit Ther Res (Special Issue on Rumination) 27: 247–259.

[50] Glynn LM, Christenfeld N, Gerin W (2007) Recreating cardiovascular responses with rumination: the effects of a delay between harassment and its recall. International J Psychophysiol 66: 135–140. 10.1016/j.ijpsycho.2007.03.018 17570551

[51] Zoccola PM, Dickerson SS, Zaldivar FP (2008) Rumination and cortisol responses to laboratory stressors. Psychosom Med 70: 661–667. 18606726 10.1097/PSY.0b013e31817bbc77

[52] Blascovich J, Tomaka J (1996) The biopsychosocial model of arousal regulation. In: Zanna MP, editor. Advances in experimental social psychology. San Diego: Academic Press. 1–51.

[53] Mischkowski D, Kross E, Bushman BJ (In press) Flies on the wall are less aggressive: Self-distancing “in the heat of the moment” reduces aggressive thoughts, angry feelings and aggressive behavior. J Exp Soc Psychol.

[54] Ekman P, Levenson R, Friesen W (1983) Autonomic nervous system activity distinguishes among emotions. Science 221: 1208–1210. 6612338 10.1126/science.6612338

[55] Tracy JL, Robins RW (2004) Putting the self into self-conscious emotions: A theoretical model. Psychol Inq 15: 103–125.

[56] Heerey EA, Keltner D, Capps LM (2003) Making sense of self-conscious emotion: Linking theory of mind and emotion in children with autism. *Emotion* 3 394–400. 14674831 10.1037/1528-3542.3.4.394

[57] Lewis M (1993) Self-conscious emotions: Embarassment, pride, shame, and guilt. In: Lewis M, Haviland-Jones J, editors. Handbook of emotion. New York: Guilford. 563–573.

[58] Orth U, Berking M, Burkhardt S (2006) Self-conscious emotions and depression: Rumination explains why shame but not guilt is maladaptive. Pers Soc Psychol Bull 32: 1608–1619. 17122174 10.1177/0146167206292958

[59] Cohen TR, Wolf ST, Panter AT, Insko CA (2011) Introducing the GASP scale: A new measure of guilt and shame proneness. J Pers Soc Psychol 100: 947–966. 21517196 10.1037/a0022641

[60] Lieberman MD, Inagaki TK, Tabibnia G, Crockett MJ (2011) Subjective responses to emotional stimuli during labeling, reappraisal, and distraction. Emotion 11: 468–480. 21534661 10.1037/a0023503 PMC3444304

[61] Conner TS, Reid KA (2012) Effects of intensive mobile happiness reporting in daily life. Soc Psychol Personal Sci 3: 315–323.

[62] Wilson TD (2005) Centerbar DB, Kermer DA, Gilbert DT (2005) The pleasures of uncertainty: Prolonging positive moods in ways people do not anticipate. J Pers Soc Psychol 88: 5–21. 15631571 10.1037/0022-3514.88.1.5

[63] Lyubomirsky S, Sheldon KM, Schkade D (2005) Pursuing happiness: The architecture of sustainable change. Rev Gen Psychol 9: 111–131.

[64] Levenson RW (2003) Autonomic specificity and emotion. In: Davidson RJ, Scherer KR, Goldsmith HH, editors. Handbook of affective sciences. New York: Oxford University Press. 212–224.

[65] Barrett LF (2006) Emotions as natural kinds? Perspect Psychol Sci 1: 28–58. 26151184 10.1111/j.1745-6916.2006.00003.x

[66] Ray RD, Wilhelm FH, Gross JJ (2008) All in the mind’s eye? Anger rumination and reappraisal. J Pers Soc Psychol 94: 133–145. 18179323 10.1037/0022-3514.94.1.133

[67] Pedersen WC, Denson TF, Goss RJ, Vasquez EA, Kelly NJ, et al (2011) The impact of rumination on aggressive thoughts, feelings, arousal, and behavior. Br J Soc Psychol 50: 281–301. 21545459 10.1348/014466610X515696

[68] Spielberger CD, Sydeman SJ (1994) State-Trait Anxiety Inventory and State-Trait Anger Expression Inventory. In: Maruish ME, editor. The use of psychological testing for treatment planning and out- come assessment. Hillsdale NJ: Lawrence Erlbaum. 292–321.

[69] Clay DL, Anderson WP, Dixon WA (1993) Relationship between anger expression and stress in predicting depression. J Couns Dev 72: 91–94.

[70] Bridewell WB, Chang EC (1997) Distinguishing between anxiety, depression, and hostility: Relations to anger-in, anger-out, and anger-control. Pers Individ Dif 22: 587–590.

[71] Matthews KA (2005) Psychological perspectives on the development of coronary heart disease. Am Psychol 60: 783–796. 16351405 10.1037/0003-066X.60.8.783

[72] Jefferson AL (2010) Cardiac output as a potential risk factor for abnormal brain aging. J Alzheimers Dis 20: 813–821. 20413856 10.3233/JAD-2010-100081 PMC3041147

[73] Tangney JP, Wagner P, Fletcher C, Gramzow R (1992) Shamed into anger? The relation of shame and guilt to anger and self-reported aggression. J Pers Soc Psychol 62: 669. 1583590 10.1037//0022-3514.62.4.669

[74] Lewis HB (1971) Shame and guilt in neurosis. New York: International Universities Press.

[75] Miller S (1985) The shame experience. Hillsdale NJ: Erlbaum.

[76] Gendron M, Lindquist K, Barsalou L, Barrett LF (2012) Emotion words shape emotion percepts. Emotion 12: 314–325. 22309717 10.1037/a0026007 PMC4445832

[77] Lindquist K, Barrett LF (2008) Constructing emotion: The experience of fear as a conceptual act. Psychol Science 19: 898–903. 10.1111/j.1467-9280.2008.02174.x PMC2758776 18947355

